# Exploitation of tolerance to drought stress in carrot (*Daucus carota* L*.*): an overview

**DOI:** 10.1007/s44154-023-00130-0

**Published:** 2023-12-11

**Authors:** Muhammad Daniyal Junaid, Zahide Neslihan Öztürk, Ali Fuat Gökçe

**Affiliations:** 1 Department of Agricultural Genetic Engineering, Ayhan Şahenk Faculty of Agricultural Sciences and Technologies, Niğde Ömer Halisdemir University, Niğde, Türkiye; 2 Department of Plant Breeding and Genetics, Faculty of Agricultural Sciences, University of The Punjab, Lahore, Pakistan

**Keywords:** Carrots, Drought, Hormonal regulation, miRNAs, Metabolites, Quality

## Abstract

Drought stress is a significant environmental factor that adversely affects the growth and development of carrot (*Daucus carota* L.), resulting in reduced crop yields and quality. Drought stress induces a range of physiological and biochemical changes in carrots, including reduced germination, hindered cell elongation, wilting, and disrupted photosynthetic efficiency, ultimately leading to stunted growth and decreased root development. Recent research has focused on understanding the molecular mechanisms underlying carrot's response to drought stress, identifying key genes and transcription factors involved in drought tolerance. Transcriptomic and proteomic analyses have provided insights into the regulatory networks and signaling pathways involved in drought stress adaptation. Among biochemical processes, water scarcity alters carrot antioxidant levels, osmolytes, and hormones. This review provides an overview of the effects of drought stress on carrots and highlights recent advances in drought stress-related studies on this crop. Some recent advances in understanding the effects of drought stress on carrots and developing strategies for drought stress mitigation are crucial for ensuring sustainable carrot production in the face of changing climate conditions. However, understanding the mechanisms underlying the plant's response to drought stress is essential for developing strategies to improve its tolerance to water scarcity and ensure food security in regions affected by drought.

## Introduction

*Daucus carota* L. is an important vegetable, grown in a cool climate and globally used for its edible taproot (Bashir et al. 2021). It is a hydrophilic and necrophile vegetable crop consisting of high nutritional compounds (Bonasia et al. 2021). High contents of anthocyanins, carotenes, and phenolics make carrots an excellent food for human nutrition requirements. This also made high consumer demand for carrots. However, carrot studies are still impoverished, and climate change poses a threat to its production (Blando et al. 2021; Kowalczyk and Kuboń 2022).

China is the largest carrot producer however, Canada and America are the biggest carrot markets, moreover Europe is the fastest-growing market in the world. About 46.3 million tons of carrots and turnips were consumed annually (FAOSTAT 2021). Carrot taproots are consumed globally as raw or cooked, in a wide range of cuisines, presence of beta-carotenes in taproots is extremely important for the human body as the human body converts it into vitamin A. It is widely used in the food processing industry and cosmetics (Kowalska et al. 2017).

Water is an important component of plant’s cell; it plays a vital role in metabolite and nutrient transportation in plant parts. However, in the absence of adequate water supply, plants exhibit high transpiration rates and disturbed metabolism (Qaderi et al. 2019). In plants, the low water stress response can comprise different biological mechanisms at the cellular or organ level during various phases of growth. Such mechanisms enhance the production of stress-responsive metabolites and proteins. Various molecular pathways are involved in stress-responsive signaling networks, transcription, regulation, and metabolism against drought stress. The plant's response to water scarcity is heavily controlled by regulatory elements i.e., transcription factors and protein kinases including mitogen-activated protein kinase (MAPK) cascades, calcium-dependent protein kinases (CDPKs/CPKs), and receptor-like kinases (RLKs) (Chen et al. 2021). Various transcription factor families including NAC, MYB, AREB/ABF, and bZIP are involved in stomatal movement and drought-related gene expression of different genes (Yao et al. 2021).

Root crops are highly sensitive to water deficit during root formation, hence water scarcity causes a negative influence on the root quality and yield of carrot plants (Junaid et al. 2023a, b). Drought stress threat is increasing at an alarming rate, every year occurrence of drought multiplies and results in severe yield and quality losses (Čimo et al. 2020). Carrot cultivation is expanding continuously, unfortunately, environmental hazards are a challenge for the growth and development of carrots (Que et al. 2019).

This review is an attempt to sum up different drought stress-related studies on carrots and to explore how the carrot plant is affected under low water stress, and its tolerance at various levels including morphological, physiological, and molecular.

## Drought stress

Abiotic stresses such as drought, heat, heavy metals, and salinity, are the most drastic abiotic stresses (Gill et al. 2015), furthermore, climate change aggravates these hazards (Junaid et al. 2021). Scarce groundwater for plant production is a grave global problem. The unavailability of moisture for plant growth is considered as drought stress and it is most severe form of abiotic stress (Naing and Kim 2021). Response to drought stress in plants is polygenic and hence complex. It is a major abiotic stress which hinders plant growth, many efforts have been done to investigate physiological and molecular mechanisms in plants under stress to improve growth and yield. However, there is still a large gap between plant yields grown under normal conditions and less water conditions.

### Drought-carrot interaction

Fluctuations in environmental conditions have remarkably influenced carrot growth and development (Huang et al. 2015; Que et al. 2019). Several studies described that water deficit is a major abiotic factor in carrot production and it negatively affects carrot yield and quality (Salter and Goode 1967; Groves and Bailey 1994; Herppich et al. 2001; Öztürk Gökçe et al. 2022; Junaid et al. 2023a, b). However, the influence of low soil moisture on carrot taproots is not clear. Our recent study showed that water scarcity severely affects the chemical composition, dry matter content, and sugar content of susceptible carrot cultivars (Junaid et al. 2023a, b). Decrease in dry matter % under drought stress is also due to inhibition of biomolecules under stress and the inability of carrot cultivars in dry matter partitioning in different parts of their roots (Polania et al. 2016). Previously it was reported that drought stress affects accumulation of reducing sugars, which plays positive role in osmoregulation under drought stress, moreover sugar contents play indirect role in carbohydrate mobilization under water scarcity (Li et al. 2020).

Older studies described that drought reduces yield; however, it was not clear which growth stages are more sensitive to drought. Furthermore, later studies indicated that carrot is not sensitive to drought at the early growth stage, however, it exhibits a decline in yield if exposed to drought stress during mid-season, which also increase the risk of other plant diseases including scab (Obidiegwu et al. 2015).

It was reported that water scarcity did not affect the chemical composition of carrot plants consistently, however, at early growth stages, it negatively influences dry matter contents. Howbeit, drought occurrence before harvest causes high dry matter and lower potassium and nitrate under sandy coarse soil, this might be due to different carrot genotypic responses (Sørensen et al. 1997). Another study also reported the role of drought stress in reduced carrot root diameters (Reid and Gillespie 2017).

**Fig. 1:**
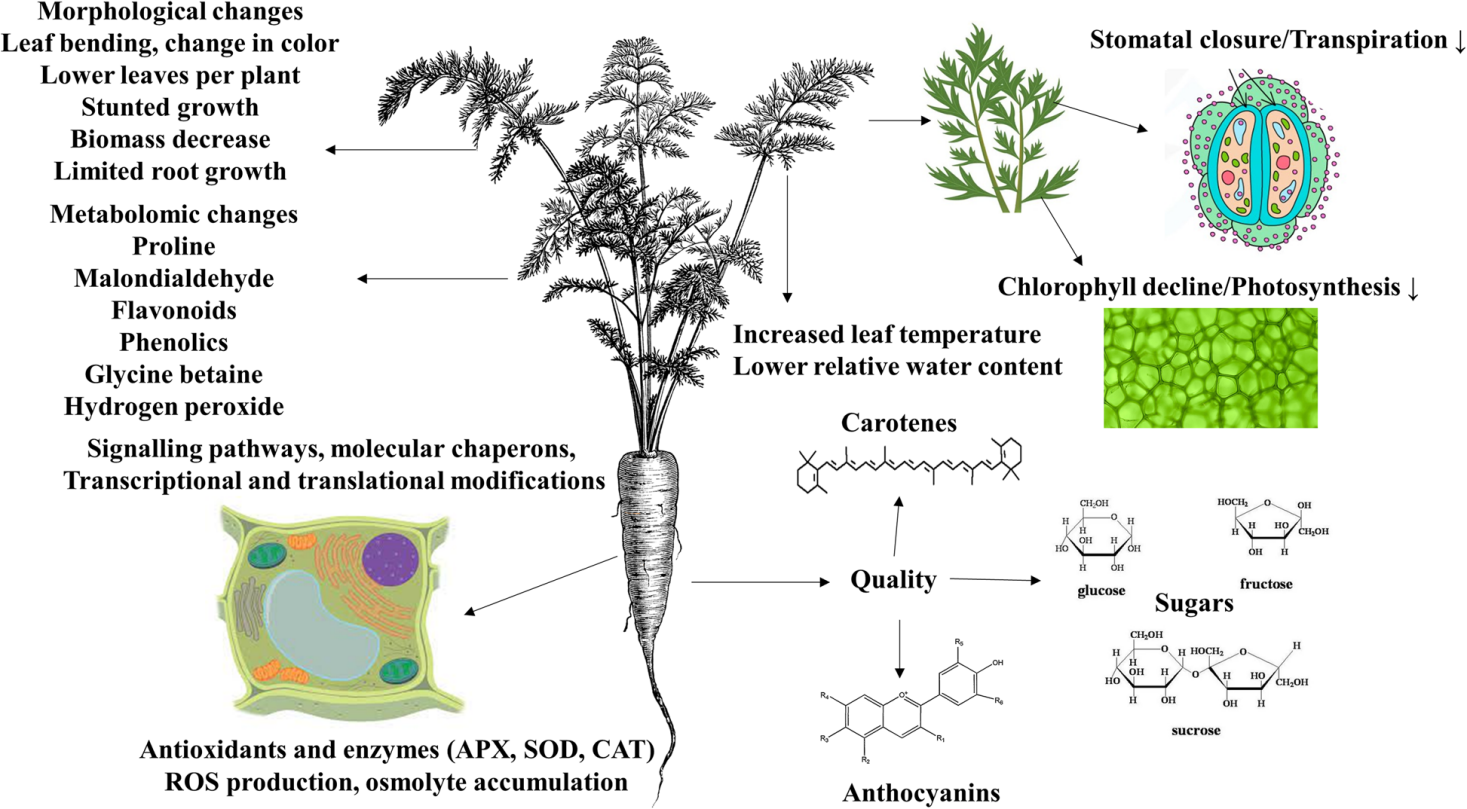
Representation of drought effects on morphological, physiological, biochemical, molecular and quality characteristics in carrot plants

Drought stress significantly causes changes in metabolite concentrations including proline, glycine betaine, and phenols in carrots (Razzaq et al. 2017). Furthermore, carotenoid contents in susceptible carrot genotypes exhibit a reduction in comparison to tolerant genotypes (Zhang et al. 2021). A general description of drought’s influence on different physiological, molecular and quality traits of carrots is presented in Fig. 1.

### Osmotic adjustment

Plants uptake water via roots when there is less water potential in plants compared to outer soil (Taiz and Zeiger 2010), such low water potential in plants is known as osmotic stress. Carrot plants exhibit osmotic adjustments when exposed to abiotic stresses including drought, such adjustments include changes in prolines, antioxidant enzymes, and glycine betaine (Öztürk Gökçe et al. 2022). Synthesis of Osmo protectants in plants enables plants to resist osmotic stress, this is also known as osmotic adjustment (Blum 2017). During drought stress, Osmo protectants decrease osmotic potential and enhance water retention in cells of other vegetables and carrots during water deficit conditions (Fang and Xiong 2015). Osmolytes in plants under low water stress include proline, mannitol, trehalose, sorbitol, glycine betaine, polyamines, etc. (Sankar et al. 2007). These compounds cause stability in the cell, however they do not affect enzyme functioning (Taiz and Zeiger 2010). Osmo-protectants control cell turgor, water gradient, and water uptake in the cytoplasmic region (Murti et al. 2007). Different studies reported osmotic adjustment in carrots under abiotic stress (Kamińska et al. 2022; Junaid et al. 2023a; Öztürk Gökçe et al. 2022). However, studies are still needed to investigate mechanisms behind osmotic regulation in carrot plants under abiotic stress.

### Hormonal regulation

Based on physiological functions and their structure, there are known regulatory hormones in plants which include jasmonic acid, ethylene, cytokinins, abscisic acid, auxins, gibberellins, and salicylic acid (Iqbal et al. 2022). These hormones are widely studied in plants (Dubois et al. 2018). Apart from these mentioned hormones, compounds derived from these hormones are also being studied as plant hormones. Hormonal regulation during development and growth in carrots are widely studied (Que et al. 2019). ABA (Abscisic acid), salicylic acid, and ethylene coordinate with each other and are important parts of the stress response mechanism in plants (Khan et al. 2015). Recent studies have accentuated that a regulatory molecule ‘melatonin’ is an important biostimulator against stress in plants, however, it is still not categorized as a phytohormone (Kanwar et al. 2018). Wang et al. 2015 performed a study in which transcriptome analysis revealed that among 4818 unigenes, 87 genes were associated with hormone related pathways. Under drought stress, ABA is the most prominent hormone which is involved with other phytohormones and signaling molecules. Study of phytohormones help in clarifying the role of hormones during root development. Evidence describes that it mobilizes biochemical defense systems in plants against stress including antioxidants, ROS (Reactive oxygen species) enzymes (Hoque et al. 2016), Heat shock proteins (Huang et al. 2015), and proline (Ashraf and Foolad 2007). Some recent hormones related studies on carrots are briefly discussed in this section.

A recent study on carrots described the role of two genes that were involved in hormone regulation during carrot storage root development (Macko-Podgórni et al. 2020), which indicates that in carrot plant hormones play a vital role in root elongation. Cell differentiation in plants can be induced by cytokinin and stem cell activity in plants might increase meristem growth, which indicates a hormonal role in plant development. Furthermore, radial root growth from vascular cambium is also controlled by certain hormones. Such hormones might be implied in carrot plants to examine their productivity under external stimuli. According to Samuolienė et al (2005), in carrots high temperature influences the biosynthesis of phytohormones, flowering time, initiation, stem elongation, and bud formation. They also explained that the application of gibberellic acid results in fast stem elongation and bud formation, moreover ABA decline in carrots might be associated with efficient flowering. In another study, a carrot cultivar ‘Flacoro’ accumulated health-promoting compounds and exhibited stress tolerance at the callus initiation stage when applied with MgO (Magnesium oxide) (Keutgen et al. 2022). It was reported that indole butyric acid (IBA) remarkably enhances growth traits in carrot plants. The same study also described that auxin accumulations in carrot plants might influence carrot taproot and lignin accumulation (Khadr et al. 2020). These studies indicate that hormones involved in carrot growth might be used to enhance carrot plant productivity in the advent of environmental stresses.

## Drought stress effects on carrot quality

Carrot root quality depends on several factors and is comprised of many essential compounds, the main contents of sugars determine the carrot's taste. Fresh carrots contain 3.5 to 10.7% of total sugar contents, whereas sucrose constitutes 56% and fructose about 18.5% of total sugar contents. Furthermore, total sugars in carrots make up 70% of total dry matter contents (Gocan et al. 2013). Drought stress reduces sugar contents in carrots and a decline in the glycolytic pathway results in cessation of growth as a result of lowered respiration (Yu 1999). Environmental stress also induces an increase in monosaccharides and disaccharides in carrots which also have an important role in osmotic protection (Gupta 2006).

Terpene contents also affect the taste and texture of carrots are also an important group of metabolites that are also categorized as bioactive compounds in carrots (Keilwagen et al. 2017). High terpene contents in carrots cause bitterness which is not liked by consumers and is a negative indicator of its economic value (Gocan et al. 2013). It has been recently reported that terpenoids might have a protective role under environmental stresses, however, the mechanism of their protective role under drought stress is still unclear (Haberstroh et al. 2018). A study was performed to investigate Terpene Synthase Gene Family in carrot, it was revealed that genes which belong to this family might be involved in resistance to abiotic and biotic stress (Keilwagen et al. 2017).

Previously, it has been stated that under abiotic stresses, there is enhanced production of anthocyanins. It has been suggested that anthocyanins might play a crucial role in mitigating photo-oxidative harm (Landi et al. 2015). Additionally, anthocyanins constitute a vital component of secondary metabolites, and they play a significant role in plant adaptation during fluctuating environmental conditions (Fraser and Chapple 2011).

## Genetic response to drought stress

Drought stress occurrence in plants results in the activation of associated genes which play actively to respond against stress. Similarly, carrot plants also show upregulation or downregulation of certain drought stress related genes i.e. proline transporters, proline dehydrogenases, catalase (Junaid et al. 2023a, b). Drought responses might be at physiological or biochemical levels which depend on plant genotype and the stress type and intensity (Gall et al. 2015). On several occasions, plants adapt to stressors, escape the drought stress, and complete their lifecycle before the normal life period. Genes that are regulated under the influence of drought stress are involved in the production of hormones, accumulation of proteins, biochemicals, and osmolytes (Mahmood et al. 2019).

## Physiological and biochemical effects of drought stress on carrots

**Fig. 2:**
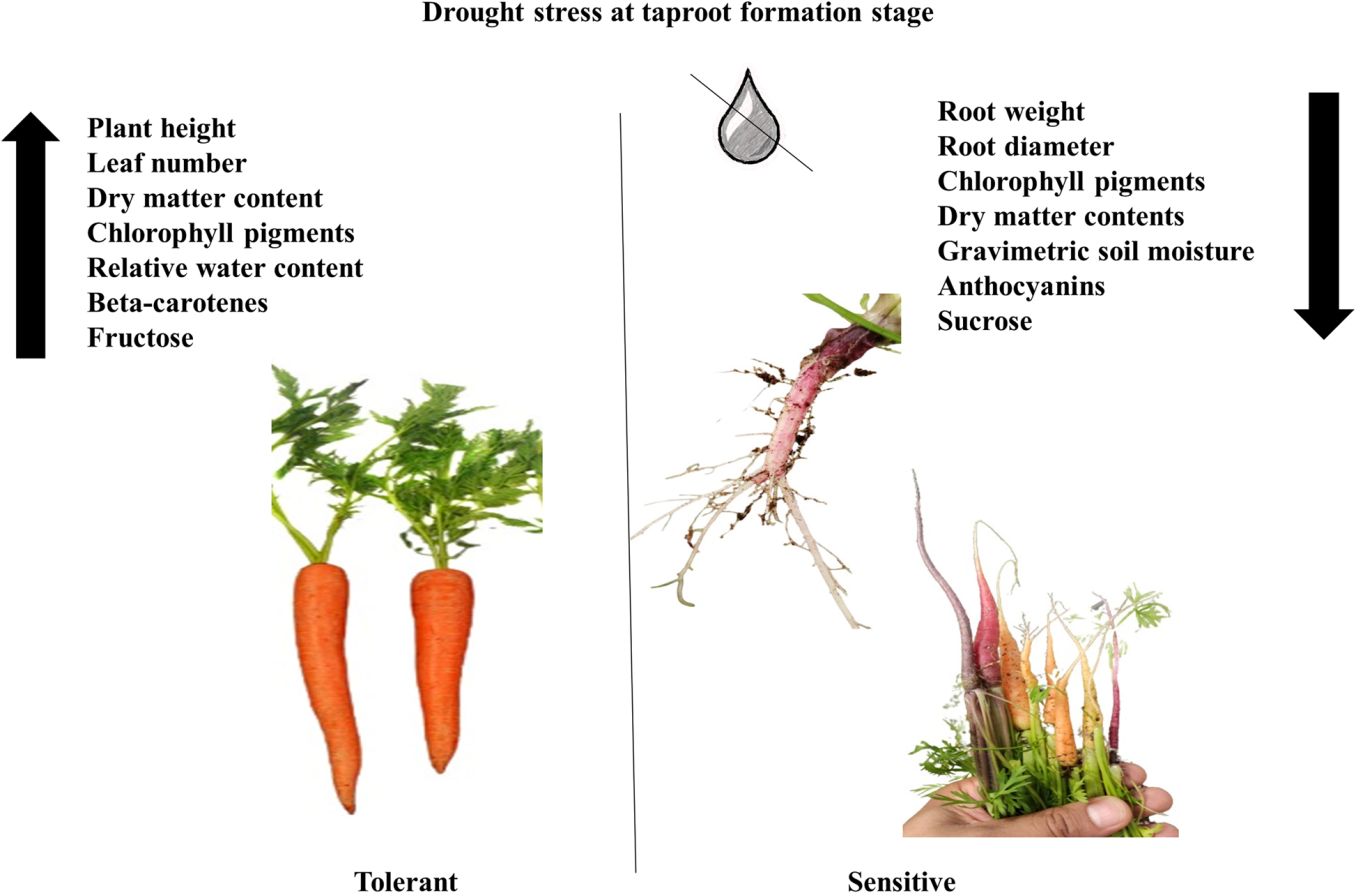
Pictorial representation interpreting the drought effect on carrot cultivars grown in controlled and drought-stress environments. Changes in different parameters under drought stress are presented (Junaid et al. 2023a, b)

Various studies on carrots explained the effect of drought stress on its physiology and biochemical properties. It was reported that the application of Gamma-aminobutyric acid (GABA) enhanced the physio-biochemical stress response of carrot plants (Bashir et al. 2021). Application of such molecules on carrot plants under drought stress might have a positive role in their response to drought stress however such studies are needed at a wider level. Carrot's biochemical machinery shows an altered response under drought stress. In our previous study, commercial carrot cultivars showed high proline levels when exposed to water deficit, however, it was reported that proline contents in carrot plants may depend on stress duration and severity. The same study also reported the role of catalase enzymes and hydrogen peroxide in carrot response to drought stress, moreover, superoxide dismutase activity also increased with enhanced oxidative stress (Junaid et al. 2023a, b). Figure 2 shows how physiological traits and sugars in carrots are influenced under drought stress.

### Glycine betaine and phenolics

Previous reports indicate that when plants are exposed to drought stress, essential metabolites such as GB (glycine betaine) and total phenolics exhibit higher levels of accumulation (Ha et al. 2012). Occurrence of drought stress causes significant increase in GB contents in carrot genotypes (Öztürk Gökçe et al. 2022). It is well-established that phenolic compounds possess the ability to scavenge reactive oxygen species (ROS) (Weidner et al. 2009) and they combine with metals to inhibit the enzyme oxidizing activity (Elavarthi and Martin 2010). Carrot plants under drought stress exhibit high phenolic contents, this is maybe due to that plants activates phenolic compounds that confer physiological stability by preventing oxidative burst and protect plants from damage of protein, lipids, and DNA structures (Öztürk Gökçe et al. 2022). Furthermore, previous studies have reported that abiotic stress can lead to both an increase and decrease in the phenolic contents within plant cells (Wrobel et al. 2005).

### Sugars

It is said that a higher accumulation of sugars in plant cells declines cell osmotic potential, so sugar concentrations in plant cells have an important role in drought stress (Granda and Camarero 2017). The sucrose contents under drought stress response also depend upon the enzymatic cleavage activity (Praxedes et al. 2006). The variations in sucrose content in different carrot cultivars could potentially be attributed to the phenomenon of sucrose hydrolysis, resulting in the conversion of sucrose into fructose and glucose (Junaid et al. 2023a, b). Drought stress may influence the enzymatic sucrose phosphate synthase which causes a decrease in fructose and glucose contents in carrots. In some studies, it has been reported that drought stress can impact the accumulation of reducing sugars, which serves a beneficial function in osmoregulation during periods of drought stress. (Praxedes et al. 2006). Moreover, sugar contents also play a role in carbohydrate mobilization under drought stress.

### Protein

Exposure to abiotic stress in plants causes alteration in their protein content accumulation which results in several modifications (Anjum et al. 2015). Carrots are rich in protein contents, when investigated it was noted that there were significant differences in protein contents between control and drought stressed carrots (Öztürk Gökçe et al. 2022). Genes that are regulated under the influence of drought stress are involved in the production of hormones, accumulation of proteins, biochemicals, and osmolytes (Cushman and Bohnert 2000). The literature described that osmotin-like proteins might be involved in stress tolerance via apo-plastic localization (Annon et al. 2014).

### Proline

Plants under stressful environments produce compatible osmolytes, one of the most important osmolytes is proline which plays an important role against stress in plants (Ozturk et al. 2021). Proline serves as a major osmoregulator, scavenges reactive oxygen species (ROS), and acts as a cellular redox buffer in plants as a response to drought stress (Dawood et al. 2019). In higher plants, proline stabilizes membrane structure, protects thylakoid membranes, scavenges reactive oxygen species, and helps conform protein structure (Hayat et al. 2012). Proline in combination with catalase, ascorbate peroxidase, and superoxide dismutase strengthens the antioxidant system of plants against external stresses (Slabbert and Kruger 2014).Proline also acts as a storage compound for carbon and nitrogen and reduces oxidative damage under stress. It also plays a vital role in signal transduction and mitochondrial regulation (Ozturk et al. 2021).

In carrot plant leaves it is reported that proline contents, phenols, and glycine betaine increase under water deficit (Razzaq et al. 2017). In another study, it was speculated that proline transporter genes express increased regulation under drought conditions and play an important role in the accumulation of proline in susceptible carrot cultivars. However, the regulation of proline-related genes depends upon the susceptibility or tolerance of carrot genotypes. Furthermore, the proline dehydrogenase gene's downregulation was also observed under water deficit (Junaid et al. 2023a, b). The same study also showed a tremendous increase in the proline content of susceptible carrot cultivars. Other studies also showed similar outcomes where the susceptible carrot genotype expressed high proline accumulation (Öztürk Gökçe et al. 2022). Increased proline might also play a role in scavenging free radicals which may be synthesized in plant chloroplasts because of abiotic stress (Rejeb et al. 2014).

### Carotenoids

Carrot plants are one of the most nutritious vegetables which have a natural tendency to produce large quantities of beta-carotene (Vitamin A precursor) (Rao and Rao 2007). Beta-carotenes are protective against ulcers, and cancer, improve the immune system, and play a photo-protectant role in cell membranes. Beta-carotene in carrots is responsible for their characteristic yellow color, however, they are susceptible to abiotic stresses including drought stress (Ahmad et al. 2019; Junaid et al. 2023a, b). Their sensitive chemical composition requires careful handling during carrot development to protect carotenes.

Antioxidant substances in plants including carotenoids also play a vital role in the reduction of ROS, alleviating drought stress and protecting plants against negative effects (Treutter 2006). They are liposoluble pigments that exist in the chromoplasts of plants and are accountable for the red, orange, and yellow colors of carrot taproots (Zhang et al. 2021). Carotenoid accumulation in higher plants is also an indicator in plants to investigate drought tolerance (Majidi et al. 2015). However, their drought resistance mechanism is complex.

The carotenoid mechanism in drought resistance is complex, in some sensitive diploid plants, it was found that carotenoids increase under drought stress in comparison with moderately tolerant plants (Parida et al. 2007). It was also reported in other vegetables that β-carotenes increase under drought stress in all genotypes irrespective of tolerance or susceptibility (Andre et al. 2009). However, in another study on carrots, susceptible cultivars exhibited decreased carotenoid contents upon exposure to drought stress (Junaid et al. 2023a, b). Several studies on different colored carrots explained that accumulation of α-carotene and lutein might be due to carotene hydroxylase genes, moreover high regulation of lycopene synthesis genes may produce lycopene in red carrot genotypes (Wang et al. 2020). It was reported that exposure to drought stress might activate the tolerance role of lutein and β-carotenes in carrot plants against water deficit (Zhang et al. 2021).

Mainly dietary vitamin A in human diet is accessible via vegetables and fruits, among vegetables carrots are the main source of provitamin A carotenoids. Beta-carotenes constitute about half of the total carotenes present in carrots, moreover, they have immense importance in the human immune system against various diseases (Grune et al. 2010).

## Molecular level studies on drought response of carrot

For any crop/vegetable, identification of the molecular basis of tolerance to stress is important. For this purpose, various researchers have performed molecular studies on carrots to investigate the molecular basis of abiotic stress tolerance in carrots. It was reported that drought tolerance might be conferred via tobacco osmotin overexpression in carrot plants (Parkhi et al. 2009). A study pointed out the resilient role of osmotin or osmotin-like proteins against abiotic stress in carrots (Annon et al. 2014). Some molecular studies also implied RNA-Seq (RNA-sequencing) to investigate the biosynthesis pathway of anthocyanins under various conditions in carrot plants (Kodama et al. 2018). Certain enzymes are involved in plant defense system against drought including catalase, ascorbate reductase, glutathione reductase, catalase, and superoxide dismutase.

It is reported that superoxide dismutase genes (*SOD*) and proline dehydrogenase genes (*PDH*) are involved in drought stress tolerance in carrot cultivars. Furthermore, proline dehydrogenase is the first enzyme involved in the proline oxidation pathway and they are associated with cellular homeostasis. The same study also described the role of catalase (*CAT*) and proline transporter genes (*PRT*) under drought stress in carrot plants, these genes are involved in the transportation of proline among organs and cells (Junaid et al. 2023a, b).

According to Wang et al. (2021), drought tolerance in carrot plants is regulated via metabolite pathways including anthocyanin, flavonoids, and cutin pathways, additionally, the transcription factor *MYB75* plays a vital role in the positive regulation of these mentioned metabolites.

### Transcriptome

Plant Genes under stress follow specific signal transduction pathways and are responsible for the plant's response via activation of the antioxidant defense system. Water scarcity alters plant gene functioning and results in new protein synthesis, late embryogenesis, and mRNAs. Genes involved in late embryogenesis are highly hydrophilic and help plants retain water in their tissues and help protect plant membranes. A transcriptomic approach to analyze gene expression, gene function, and molecular marker development is widely used in human, animal, and plant biology. In carrot plants, transcriptome analysis is widely implied to investigate novel genes and reveal new markers. Transcriptomic sequencing revealed 114 polymorphic SSRs (simple sequence repeats) and 20.058 SNPs (single nucleotide polymorphisms) in carrots (Que et al. 2019), however, the first web-based transcriptomic database for carrots was developed by Xu et al. (2014). RNA-seq analysis of purple carrot genotypes revealed differential transcriptomic responses to drought stress which indicated their tolerance level, 514 genes were found to be upregulated under drought stress in a purple carrot genotype however 622 genes were upregulated under similar circumstances in another carrot genotype (P1129). It was described that MYB-48 transcription factor, F-box protein, ferric reduction oxidase, and ABA-induced somatic embryo genes express up-regulation under water deficit (Öztürk Gökçe et al. 2022). Another study found that carotenoid and xanthophyll contents in carrots might be under the control of *DcLBCY*, *DcLECY*, and *DcZEP1* genes (Ma et al. 2018). Root transcriptomics investigation on five wild and six cultivated carrots revealed that domestication of carrots is influenced by the upregulation of water channel protein, carotenoid binding proteins, and silencing of allergen proteins. Moreover, a transcriptomic study on four different growth stages in carrots showed differential regulation of 4818 genes. Among them, 87 genes were associated with hormonal pathways. It was also revealed that root development depends upon the signaling and biosynthesis of phytohormones (Que et al. 2019).

Several transcriptomic studies revealed interesting mechanisms and genes (*NCED, SOD, PRT2, DcTLP, DcHsfs, DcAL4, DcBCH1, FRO, WRKY*) involved in different processes in carrot plants including stress tolerance (Zhang et al. 2021; Li et al. 2021; Junaid et al. 2023a, b; Huang et al. 2015; Nan and Gao 2019; Quiroz-Iturra et al. 2022) (Table 1). However, more transcriptomic investigations are needed to understand water scarcity influence on carrots and their behavior upon exposure to drought stress at different developmental stages. Researchers agree that genes involved in regulation are not only linked with transcription but are also involved in post-transcriptional modifications, translation, and transport (Gorospe et al. 2011). High throughput sequencing in plants has expanded plant molecular knowledge about RNA and gene regulation mechanisms. There are RNAs in nature that are non-coding, small (21–24 nucleotides), endogenous, and highly conserved across species which are involved in the regulation of plant growth, signaling, development and activate response to external environmental factors including drought stress (Pagano et al. 2021). Such RNAs are known as microRNAs (miRNAs), several studies indicate that miRNAs play an active role in response to abiotic stress in different plants including peanuts (Ren et al. 2021), sunflower (Liang et al. 2020), pigeon peas (Buch et al. 2020), potato (Liao et al. 2021), Arabidopsis (Fasani et al. 2021) and tea (Zhu et al. 2021).

**Table 1:** List of potential genes involved in abiotic stress regulation in carrots

Sr no	Genes	Stress type	Source
1	beta-carotene hydroxylase, chloroplastic-like (*DcBCH1)*	Drought, salinity	Li et al. 2021
2	ALFIN-like transcription factor* (DcAL4)*	Drought	Quiroz-Iturra et al. 2022
3	ABRE binding protein* (DcAREB3)*	Drought, salinity	Quiroz-Iturra et al. 2022
4	Phytoene synthase-2* (DcPSY2)*	Salinity	Quiroz-Iturra et al. 2022
5	Lycopene β-cyclase *(DcLCYB)*	Drought	Zhang et al. 2021
6	9-cis-epoxycarotenoid dioxygenases* (NCED, DcNCED1, DcNCED2)*	Drought	Zhang et al. 2021
7	Proline transporter-1* (PRT1)*	Drought	Junaid et al. 2023a, b
8	Copper-Zinc Superoxide dismutase* (Cu–Zn/SOD)*	Drought	Junaid et al. 2023a, b
9	MYB Proto-oncogene 75* (MYB-75)*	Drought	Öztürk Gökçe et al. 2022
10	Ethylene-responsive transcription factor* (RAP2-3)*	Drought	Öztürk Gökçe et al. 2022
11	Ferric reduction oxidase *(FRO)*	Drought	Öztürk Gökçe et al. 2022
12	Catalase* (CAT)*	Drought	Junaid et al. 2023a, b
13	Proline transporter-2* (PRT2)*	Drought	Junaid et al. 2023a, b
14	Thau- matin-like protein *(DcTLP)*	Drought	Jung et al. 2005
15	Heat shock factors* (DcHsfs)*	Heat	Huang et al. 2015
16	*WRKY* transcription factors* (WRKY)*	Mechanic	Nan and Gao 2019

Owing to the important role of miRNAs they are important factors to investigate for resilient and high-yielding crop/vegetable development. Until 2004 there were no miRNAs identified in carrot plants and miRNA-related studies were scarce however, 17 miRNAs were identified in a study on carrots that belonged to different families, among them 6 miRNAs were stress-associated, and others were involved with the metabolism and transcription factors (Barozai 2013; Que et al. 2019). A list of some stress-associated genes in carrots is given in Table 1.

### Metabolome

Plant metabolome refers to all small molecule chemicals present in it; studies have described the effect of drought stress on carrot metabolome. Abiotic stress triggers metabolic regulation in plants to safeguard plant cell's osmotic potential, during this process profiling of metabolites in plants is helpful to investigate changes in plant metabolome under stress. Water plays an important role in metabolite and nutrient transportation in plants. Changes in their accumulation result in altered metabolite production (Kumar et al. 2021).

It is reported that water deficiency causes high proline accumulation, GB, and phenols in carrot leaves (Razzaq et al. 2017). In another study drought tolerant carrot genotypes were screened based on their membrane thermostability (Aamir et al. 2019).

In some plants, late embryogenesis abundant proteins, heat shock proteins, and other protectant proteins play important role against drought stress (Priya et al. 2019). Under osmotic stress such proteins are vital, heat shock proteins are also capable of binding, folding, changing, and degrading other proteins, and osmotins protect cells against metabolic changes (Yang et al. 2021). Some biochemical traits influenced by drought stress in carrots are given in Table 2.

**Table 2:** Recent studies on biochemicals in carrot plants influenced by water scarcity

Sr	Compounds	Stress effect on biochemicals	Source
1	Proline	Increases	Öztürk Gökçe et al. 2022, Junaid et al. 2023a, Razzaq et al. 2017
2	Malondialdehyde	Increase	Junaid et al. 2023a, b, Öztürk Gökçe et al. 2022, Hameed et al. 2021, Razzaq et al. 2017
3	Catalase	Increase	Junaid et al. 2023a, b, Razzaq et al. 2017, Öztürk Gökçe et al. 2022
4	Superoxide dismutase	Increase	Öztürk Gökçe et al. 2022, Junaid et al. 2023a, b, Razzaq et al. 2017
5	Flavonoid	Increase	Öztürk Gökçe et al. 2022
6	Phenolics	Increase	Öztürk Gökçe et al. 2022, Razzaq et al. 2017
7	Chlorophyll	Decrease	Öztürk Gökçe et al. 2022, Hameed et al. 2021, Razzaq et al. 2017
8	Anthocyanins	Increase	Öztürk Gökçe et al. 2022
9	Leaf carotenoids	Decrease	Öztürk Gökçe et al. 2022
10	Flavonoids	Increase	Öztürk Gökçe et al. 2022
11	Glycine betaine	Increase	Razzaq et al. 2017, Öztürk Gökçe et al. 2022, Hameed et al. 2021
12	Hydrogen peroxide	Increase	Öztürk Gökçe et al. 2022, Junaid et al. 2023a, Hameed et al. 2021, Razzaq et al. 2017
13	Antioxidants	Increase	Öztürk Gökçe et al. 2022
14	Carotenes	Variable	Zhang et al. 2021
15	Ascorbate peroxidase	Increase	Öztürk Gökçe et al. 2022, Hameed et al. 2021

## Way forward?

To develop stress-tolerant carrot varieties, it is necessary to overcome various genetic, environmental, and practical constraints. These constraints are challenging, but they are important to consider for successful development of stress-tolerant carrot genotypes. Various techniques in crop/vegetable improvement are being utilized in the modern era, including the identification of tolerant traits in a genotype and incorporating them via different approaches. To develop stress-tolerant carrot cultivars, identification of stress-tolerant genes in carrot germplasm is an important approach. Then, gene expression analysis of such potential genes along different carrot genotypes could yield tremendous results. Gene expression studies have become popular to investigate metabolic and signaling pathways that control various developmental and cellular processes. Real-time qPCR is commonly used to measure relative levels of gene expression. Implication of plant breeding methods has great potential to speed up stress-tolerant plant development. In the modern era, the development of transgenic plants to incorporate desired traits in plants has caught tremendous attention from researchers. however, it might not be completely effective to produce drought tolerant carrot genotypes, as it requires complex and exorbitant laboratory methods, equipment and its success rate is truncated. Marker-assisted breeding has been instrumental for the past decades in the development of new vegetable and crop cultivars. Moreover, in-vitro plant propagation of plants is also a useful tool in vegetable breeding. Recently, the use of gene editing technologies such as CRISPR/Cas system for incorporating desired traits in plants has become popular. For genetic investigation of drought tolerance in carrots, the use of haploids, double haploids, gene mapping, and QTLs (Quantitative trait loci) might also be beneficial. There is still a significant research gap in understanding drought effects and its mechanisms in carrot crop and developing tolerant carrot cultivars. Therefore, it is needed to incorporate both conventional and modern techniques to acquire fruitful results.

## Data Availability

Not applicable.

## Abbreviations

*miRNAs*: MicroRNAs
*ABA*: Abscisic acid
*ROS*: Reactive oxygen species
*MgO*: Magnesium Oxide
*IBA*: Indole butyric acid
*GABA*: Gamma-aminobutyric acid
*GB*: Glycine betaine
*RNA-seq*: RNA sequencing
*CAT*: Catalase
*SOD*: Superoxide dismutase
*PDH*: Proline dehydrogenase
*SSR*: Simple sequence repeats
*SNP*: Single nucleotide polymorphism
*QTL*: Quantitative trait loci
